# Telemetric Assessment and Comparison of Regional Colonic Metabolic Activity in Ambulant Healthy Individuals Using pH and Gas‐Sensing Wireless Motility Capsules

**DOI:** 10.1111/apt.70345

**Published:** 2025-08-26

**Authors:** Phoebe A. Thwaites, Chu K. Yao, Jasjot Maggo, Francis C. Parker, Emma P. Halmos, Rebecca E. Burgell, Jane G. Muir, Daniel So, Kourosh Kalantar‐Zadeh, Richard B. Gearry, Peter R. Gibson

**Affiliations:** ^1^ Department of Gastroenterology, School of Translational Medicine Monash University and Alfred Health Melbourne Victoria Australia; ^2^ University of Otago Christchurch New Zealand; ^3^ Faculty of Engineering, School of Chemical and Biomolecular Engineering The University of Sydney Camperdown New South Wales Australia

**Keywords:** carbon dioxide, colonic fermentation, colonic transit time, gas profiles, hydrogen, intestinal physiology, microbiome, wireless motility capsule

## Abstract

**Background:**

Ingestible wireless motility capsules enable locoregional quantification of luminal pH and concentrations of hydrogen and carbon dioxide in the human colon.

**Aim:**

To evaluate these measures in the colon of healthy adults.

**Methods:**

Gas‐sensing and pH‐sensing wireless motility capsules were ingested tandemly and repeatedly over time. Measurements were analysed and compared in proximal and distal segments of the colon.

**Results:**

In paired datasets from 37 participants, colonic pH rose from a median 6.3 (IQR 5.8–6.9) proximally to 7.0 (6.6–7.2) distally (*p* < 0.001). Concentrations of carbon dioxide rose in nearly all participants from 12.7 (9.1–18.6) proximally to 18.8 (11.9–28.1) %.h/h distally (*p* < 0.001) with a positive correlation between proximal and distal colon (*r* = 0.76; *p* < 0.001). Hydrogen concentrations showed widely varied proximal‐to‐distal gradients with an increase in 69% of participants, but no correlation between proximal and distal colon measures. No significant correlations between colonic pH, hydrogen concentrations, and carbon dioxide concentrations were observed. Comparison of hydrogen and carbon dioxide concentrations between tandem gas‐sensing capsules by Bland–Altman analysis (*n* = 24) showed minimal (< 1.2%) bias for both measures, and gas metrics on repeat ingestion were similar (*n* = 20). However, there was greater variance in the distal colon.

**Conclusions:**

Both wireless motility capsules evaluate different yet complementary aspects of colonic fermentation. Carbon dioxide concentrations that most likely reflect overall microbial metabolic activity were consistently greater distally, while proximal‐to‐distal gradients in hydrogen concentrations varied, likely due to inter‐subject variations in dietary carbohydrate and/or methanogenesis. Luminal pH poorly reflects carbohydrate fermentation in the distal colon.

**Trial Registration:**

ACTRN12619001219178 and ACTRN12622000422729

## Graphical abstract

Evaluation of the novel gas‐sensing wireless motility capsules in healthy cohorts demonstrated that, in addition to quantifying regional transit times, hydrogen and carbon dioxide concentrations provide unique and reproducible insight into locoregional colonic physiology pertaining to microbial metabolic activity and fermentation.

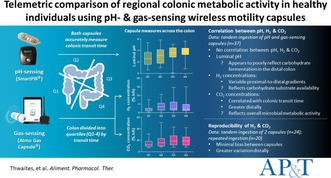

## 1 Introduction

The luminal environment in the gastrointestinal tract contains a vast array of products, derived from dietary ingestion and digestion, luminal secretions, chemical reactions, and microbial metabolic activity. Microbial metabolic activity varies according to the type and relative abundance of microbes present and is influenced by the locoregional microenvironment; it includes schemes of aerobic respiration and anaerobic fermentation [1, 2]. Many metabolites are volatile and include hydrogen (which is almost exclusively due to fermentation of carbohydrates), carbon dioxide (which may be derived from respiration, fermentation and other microbial pathways, including diffusion from the circulation and chemical reactions), methane (derived exclusively from the activity of anaerobic archaea) and short‐chain fatty acids [3, 4, 5, 6, 7]. The types and amounts of these by‐products differ across individuals, creating a unique gut metabolic profile or signature, which is dynamic both in time and space.

The development of ingestible capsules capable of sensing and telemetrically transmitting information about concentrations of volatile small molecules within the colon or concentration of other analytes, such as pH, has enabled locoregional assessment of capsule transit and potential mapping of metabolites along the gastrointestinal tract [8, 9]. Wireless motility capsules offer a safe and convenient alternative to other methods, which are often invasive, poorly standardised, and of limited sensitivity, and may require periprocedural preparation or manipulation of the gut [10, 11, 12, 13]. Ingestible capsules, on the other hand, are capable of longitudinal, real‐time assessment of the whole gastrointestinal tract in an ambulant person.

Of wireless motility capsules, a pH‐sensing capsule (SmartPill) captures luminal pH, as well as temperature and pressure, while a gas‐sensing capsule (Atmo Gas Capsule) takes measurements of the concentrations of hydrogen and carbon dioxide/methane, along with other measures to help identify gastrointestinal landmarks as previously published [10, 14]. Since the ability to regionally localise both of these wireless motility capsules in the gastrointestinal tract has been validated [10, 14, 15], both have the potential to provide unique insight into the locoregional luminal milieu, as, for example, a window into microbial metabolic activity in humans.

In order to understand perturbations of fermentation and other metabolic activities that might occur in disease states or with therapeutic manipulations, normal patterns need to be defined. Furthermore, the stability of the data over time also needs to be assessed, as wide fluctuations may limit the usefulness of the wireless motility capsules as a tool to evaluate such function. Hence, by the use of data from tandem and repeat ingestion involving the gas‐sensing and pH‐sensing capsules [10], the current study aimed first to define the patterns of colonic gas concentrations in healthy individuals without gastrointestinal symptoms; second, to compare luminal pH with other markers related to fermentation/normal gut physiology; third, to define the intra‐individual variance of data; and finally, to determine the stability of such patterns in the individual during repeated evaluation over time.

## 2 Materials and Methods

### 2.1 Participants

Healthy volunteers without gastrointestinal disease and aged 18–65 years from two clinical trials were studied. In the first trial conducted across Australia and New Zealand [10], subjects ingested two wireless motility capsules in tandem: either a pH‐sensing capsule (SmartPill, Medtronic, Minneapolis, MN) with a gas‐sensing capsule (Atmo Gas Capsule, Atmo Biosciences Ltd., Melbourne, Australia); or two gas‐sensing capsules. In the second trial, subjects ingested the gas‐sensing capsule on two separate occasions. A subset of subjects participated in both trials and so ingested the gas‐sensing capsule on multiple occasions. Volunteers were screened by clinical investigators prior to enrolment. They were excluded if they had regular gastrointestinal symptoms suggestive of a disorder of gut‐brain interaction; known structural gastrointestinal disease; difficulty swallowing; previous abdominal surgery, history of radiation enteritis, gastric bezoar, or bowel obstruction; presence of a medical condition that may alter gastrointestinal motility (including endocrine and neurological); an implantable device, such as a pacemaker; use of antibiotic, prebiotic, or probiotic in the previous 4 weeks; use of proton pump inhibitor or H_2_‐receptor antagonist in the previous 7 days; prokinetic use in the preceding 48 h; body mass index > 35 kg/m^2^ (Australian cohort) or > 27 kg/m^2^ (New Zealand cohort); or current pregnancy or breastfeeding.

### 2.2 Protocol

The protocols were approved by the Monash University Human Research Ethics Committee and the Northern B Health and Disability Ethics Committee and were registered on the Australian New Zealand Clinical Trials Registry (Registration numbers ACTRN12619001219178 and ACTRN12622000422729).

In the first trial, after an overnight fast of at least 8 h, participants consumed a 260‐kcal nutrient bar (SmartBar, Medtronic) with a glass of water, followed by tandem ingestion of two capsules that had been activated and calibrated according to the manufacturers' instructions. One pH‐sensing and one gas‐sensing capsule were ingested one after the other (i.e., in tandem) according to computer‐generated randomisation. Following capsule ingestion, participants fasted for a further 6 h and then resumed their habitual diet. They were permitted to consume at least 50 mL of water at ambient temperature every 30 min for the first 2 h of the study. Strenuous activity and smoking were discouraged. Participants were requested to keep the data receivers within 1.5 m of their body for the duration of the study and recorded bowel movements and symptoms until excretion of capsules. Plain abdominal imaging was performed if capsule excretion could not be confirmed using the respective devices. A subset of these participants tandemly ingested two gas‐sensing capsules using the same protocol. The second trial investigated an alternative nutrient bar in a randomised cross‐over design in which subjects ingested one gas‐sensing capsule on two separate occasions after consuming one of two bars—Atmo Motility Bar or SmartBar—approximately matched for energy (1064 vs 1073 kJ, respectively) and nutrients (protein 14.8 vs 15.1 g; fat 2.9 vs 2.2 g; carbohydrates 40.2 vs 42.2 g; fibre 2.8 vs 2.7 g) following the protocol described above. No statistical difference in regional transit times was observed between ingestions using the bars. For example, median (interquartile range) colon transit time was 23.5 (22.7–24.3) h for the Atmo Motility Bar compared with 24.4 (23.5–28.3) h for the SmartBar (*n* = 10; *p* = 0.084, Wilcoxon signed‐rank test) with a coefficient of variation of 7.9% (Dr Daniel So, unpublished data).

### 2.3 Evaluation of Capsule Data

For both capsules, colonic entry was defined by passage through the ileocaecal junction. This was identified by characteristic pH changes on the pH‐sensing capsule—specifically, a fall of ≥ 1 pH unit distal to gastric emptying (by at least 30 min) and lasting for at least 10 min [10]. For the gas‐sensing capsule, gastric emptying was identified primarily by a steep drop in oxygen level with corresponding rises in hydrogen and carbon dioxide concentrations. Colonic exit was defined by capsule excretion, recognised by a temperature drop or loss of signal corresponding with bowel movement for both capsules.

Colonic transit time was calculated for each capsule as the time between the ileocaecal junction and body exit (i.e., capsule excretion). Data were included for analysis if the colonic transit time was able to be calculated for paired capsules ingested. The output generated from the thermal conductivity detectors of the gas‐sensing capsule was twofold. First, hydrogen concentrations were calculated (sensitivity > 1%) as previously described [11]. Second, the concentration of carbon dioxide was corrected for hydrogen concentration since 0.2% hydrogen is detected by this sensor. Methane is also detected by this sensor at a similar molar concentration (i.e., 1% carbon dioxide = 1% methane), but is unable to be distinguished by the sensor (Dr Kyle, Berean, Atmo Biosciences, personal communication). Since the formation of one molecule of methane requires one molecule of carbon dioxide, the composite total was referred to as ‘carbon dioxide’. However, for each molecule of methane, 2 molecules of hydrogen are consumed, which may reduce the hydrogen concentrations.

The distribution of pH and gases was assessed across the colon overall and divided into quartiles based on colonic transit time, with quartile 1 (Q1) representing the proximal colon and Q4 the distal colon. Gas measurements from the gas‐sensing capsules were included if ≥ 50% of data was available for the corresponding segment. Data loss and excluded numbers are shown in Table S1. Luminal pH was expressed as median. Gas concentrations were expressed as time‐normalised area‐under‐the‐curve (AUC). This measure was used to minimise bias introduced by transit time and data loss: time‐normalisation reduced bias from differences in colonic transit time; and, owing to the discreet measures from the gas‐sensing capsule, AUC calculations are effective in interpolation when data are missing. The ratio of Q1:Q4 for pH and gas concentrations were also calculated, since these unequivocally represent the proximal and distal colon, respectively.

### 2.4 Statistical Analysis

Data were described using median (interquartile range, IQR) unless otherwise described. Results across pH, hydrogen, and carbon dioxide metrics were assessed via pair‐wise differences between Q1 and Q4; these were compared using Wilcoxon signed‐rank tests and Spearman correlations. For tandem and repeat studies of the gas‐sensing capsules, paired data were compared by Spearman correlation, and Bland Altman graphs were plotted to calculate the bias and coefficients of variation. A *p*‐value ≤ 0.05 was considered statistically significant. Data were analysed using Stata 18 (StataCorp LLC, College Station, TX) and GraphPad Prism (version 10.4.0; GraphPad Software, San Diego, CA).

## 3 Results

### 3.1 Participants

In the first trial, 50 participants (19 male; mean age 33 [range: 21–65] years; mean body mass index (BMI) 24 [range: 16–34] kg/m^2^) tandemly ingested the pH and gas‐sensing capsules (i.e., one immediately after the other) of whom 37 provided paired colonic datasets suitable for analysis. Twenty‐four subjects (13 male; aged 31 [23–56] years; BMI 24 [20–32] kg/m^2^) completed the tandem ingestion of the gas‐sensing capsules, of whom 20 provided paired colonic datasets suitable for analysis. Across these cohorts and including 10 subjects who participated in a second trial, there was a total of 21 subjects (16 male; aged 34 [22–60] years; BMI 25 [19–32]) who ingested the gas‐sensing capsule on two separate occasions a median 28 (9–77) days apart, with colonic data from all subjects suitable for analysis.

### 3.2 Tandem Ingestion of pH‐ and Gas‐Sensing Wireless Motility Capsules

#### 3.2.1 Colonic pH

**FIGURE 1:**
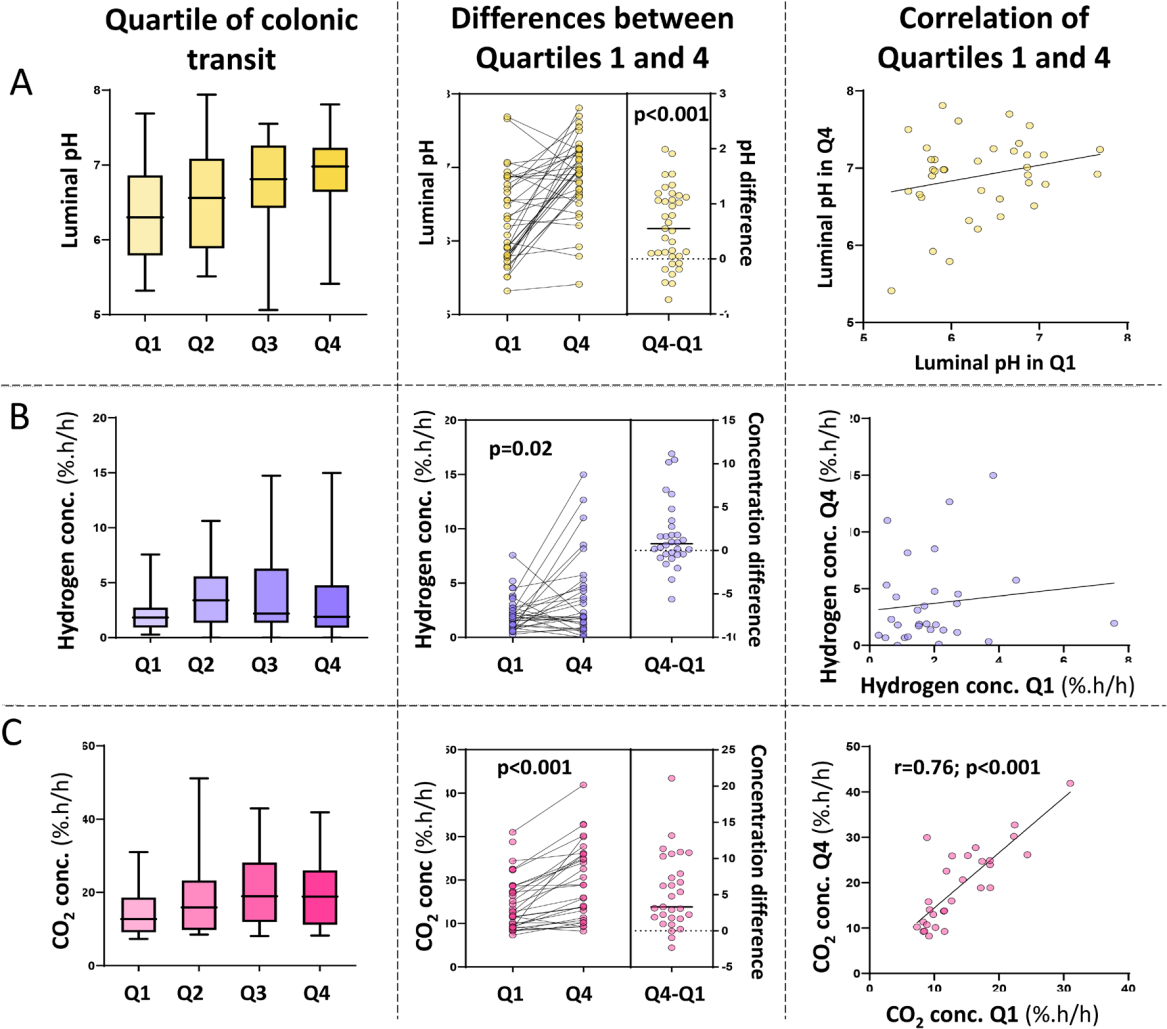
Quartile analysis (according to colonic transit) of (A) colonic pH, (B) hydrogen concentrations and (C) carbon dioxide (CO_2_) concentrations obtained from tandemly‐ingested pH‐ and gas‐sensing wireless motility capsules. Results are shown as median, IQR and range for values across the colon and differed across the quartiles (Friedman's test) for pH (*p* < 0.0001), hydrogen (*p* = 0.013) and carbon dioxide (*p* < 0.0001). There were differences for the three measures between Q1 and Q4 (Wilcoxon signed‐rank tests test). Spearman correlations are shown between paired results from Q4 and Q1.

**FIGURE 2:**
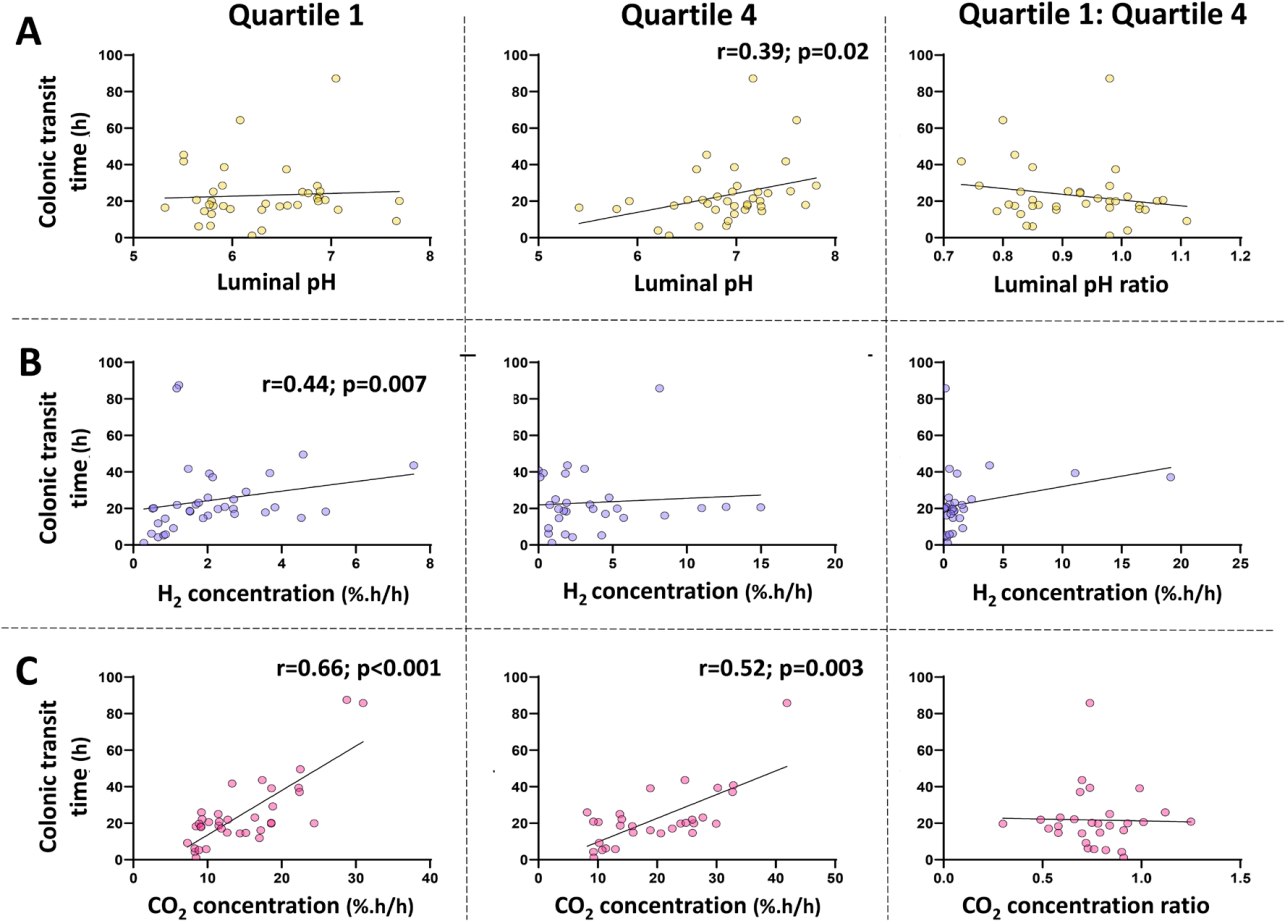
Relationship of the colonic transit time with (A) median pH, (B) median hydrogen concentrations and (C) median carbon dioxide concentrations within the first (Quartile 1) and last (Quartile 4) quartile of colonic transit, and their ratios of Quartile 1: Quartile 4. Spearman correlations were performed and only statistically significant (*p* ≤ 0.05) relationships are shown.

Median colonic transit time as measured by pH‐sensing capsule was 20 (IQR 16–25) h. The median pH across the entire colon was 6.5 (6.3–7.0) and across the quartiles is shown in Figure 1A. Individual results are shown in Figure S1. The pH of Q1 was 6.3 (5.8–6.9) compared with 7.0 (6.6–7.2) in Q4 (*p* < 0.001) with a Q1:Q4 ratio of 0.93 (0.84–0.99). Higher pH in Q4 relative to Q1 was observed in 29 (78%) of subjects. No significant correlation was observed between Q1 and Q4 on Spearman correlation (*r*_*s*_ = 0.17, *p* = 0.31). As shown in Figure 2A, the colonic transit time correlated with the pH in Q4 (*r*_*s*_ = 0.39, *p* = 0.02), but not with pH in Q1 (*r*_*s*_ = 0.12, *p* > 0.05) nor with the Q1:4 pH ratio (*r*_*s*_ = −0.21, *p* > 0.05).

#### 3.2.2 Hydrogen Concentrations

Median colonic transit time as measured by the gas‐sensing capsule was 20 (15–33) h. The median colonic hydrogen concentration across the entire colon was 2.6 (1.5–4.9) %.h/h and those across the quartiles are shown in Figure 1A. Individual results are shown in Figure S1. Hydrogen concentrations were 1.9 (0.9–4.8) %.h/h in Q4 compared with 1.8 (0.9–2.7) %.h/h in Q1 (*p* = 0.02; Figure 1B). In participants with valid data for both Q1 and Q4, higher hydrogen in Q4 relative to Q1 was more commonly observed (20 [69%] participants); the Q1:Q4 ratio observed overall was 0.7 (0.3–1.5). A moderate, positive correlation between colonic transit time and Q1 hydrogen concentration (*r*_*s*_ = 0.44; *p* = 0.007), but not Q4 (*r*_*s*_ = 0.00; *p* > 0.05) or the Q1:Q4 ratio (*r*_*s*_ = 0.25; *p* > 0.05) (Figure 2B).

#### 3.2.3 Carbon Dioxide Concentrations

The median concentration of carbon dioxide across the entire colon was 18.1 (10.3–23.8) %.h/h. Quartile activities are shown in (Figure 1C) and across the quartiles are shown in Figure 1A. Individual results are shown in Figure S1. Carbon dioxide concentrations were 18.8 (11.9–28.1) %.h/h in Q4 compared with 12.7 (9.1–18.6) %.h/h in Q1 (*p* < 0.001), reflecting individual observations where carbon dioxide concentration was higher in Q4 compared with Q1 in 26 (90%) participants. A strong, positive correlation was observed between carbon dioxide concentrations in Q1 and Q4 (*r*_*s*_ = 0.76; *p* < 0.001). There were moderate, positive correlations between colonic transit time with carbon dioxide concentrations in Q1 (*r*_*s*_ = 0.66; *p* < 0.001) and Q4 (*r*_*s*_ = 0.52; *p* = 0.003), but not the Q1:Q4 ratio (*r*_*s*_ = −0.02; *p* > 0.05) (Figure 2C).

### 3.3 Relationship of Carbon Dioxide to Hydrogen Concentrations and Colonic Transit

**FIGURE 3:**
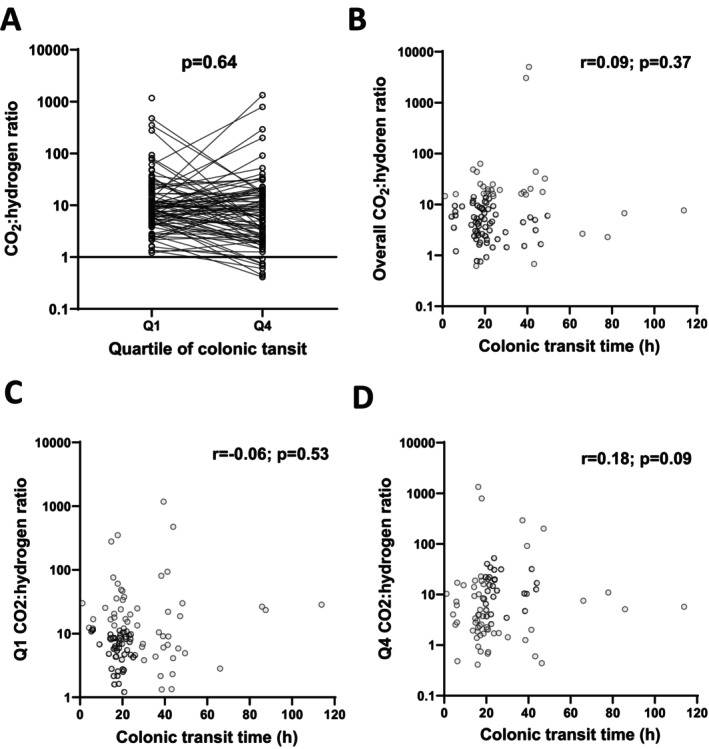
Carbon dioxide: Hydrogen ratios for the entire dataset showing (A) The relationship between the ratios in Q1 and Q4, compared with Wilcoxon test; and the relationship of colonic transit time with the ratios (B) overall, (C) in quartile 1 (Q1), and (D) in quartile 4 (Q4). The results of Spearman correlations are shown.

The carbon dioxide: hydrogen concentration ratios were 20.3 (16.3–36.0) across the colon and did not differ across the colonic quartiles (Figure S1). The ratios did not differ in Q1 compared with those in Q4 (*p* = 0.064; Figure 3A). An increasing proximal‐distal ratio was noted in 48% of participants. The carbon dioxide: hydrogen concentration ratio for the entire colon, and in Q4 and Q1, did not correlate with the colonic transit time (Figure 3B–D).

### 3.4 Comparisons Within Colonic pH and Gas Concentrations

**FIGURE 4:**
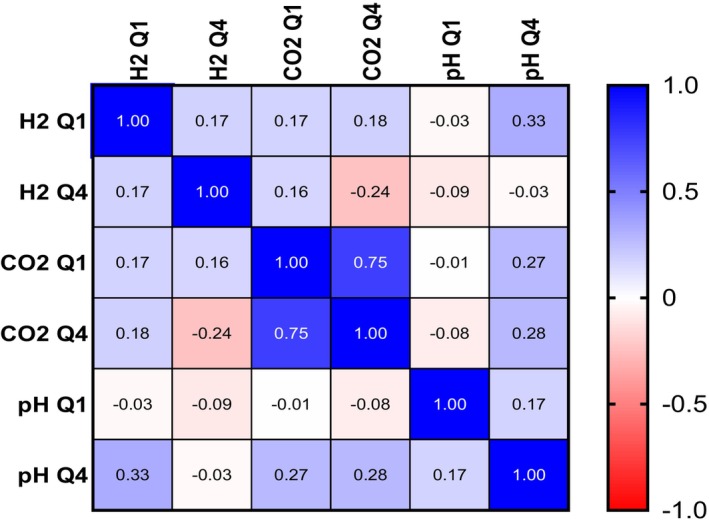
Spearman correlation matrix of pH, hydrogen (H2) concentration and carbon dioxide (CO_2_) concentration across quartiles 1 (Q1) and 4 (Q4) of colonic transit via tandemly‐ingested pH‐ and gas‐sensing wireless motility capsules.

No significant correlations between colonic pH, hydrogen, and carbon dioxide concentrations were observed (Figure 4).

### 3.5 Tandem Ingestion of Gas‐Sensing Capsules

**FIGURE 5:**
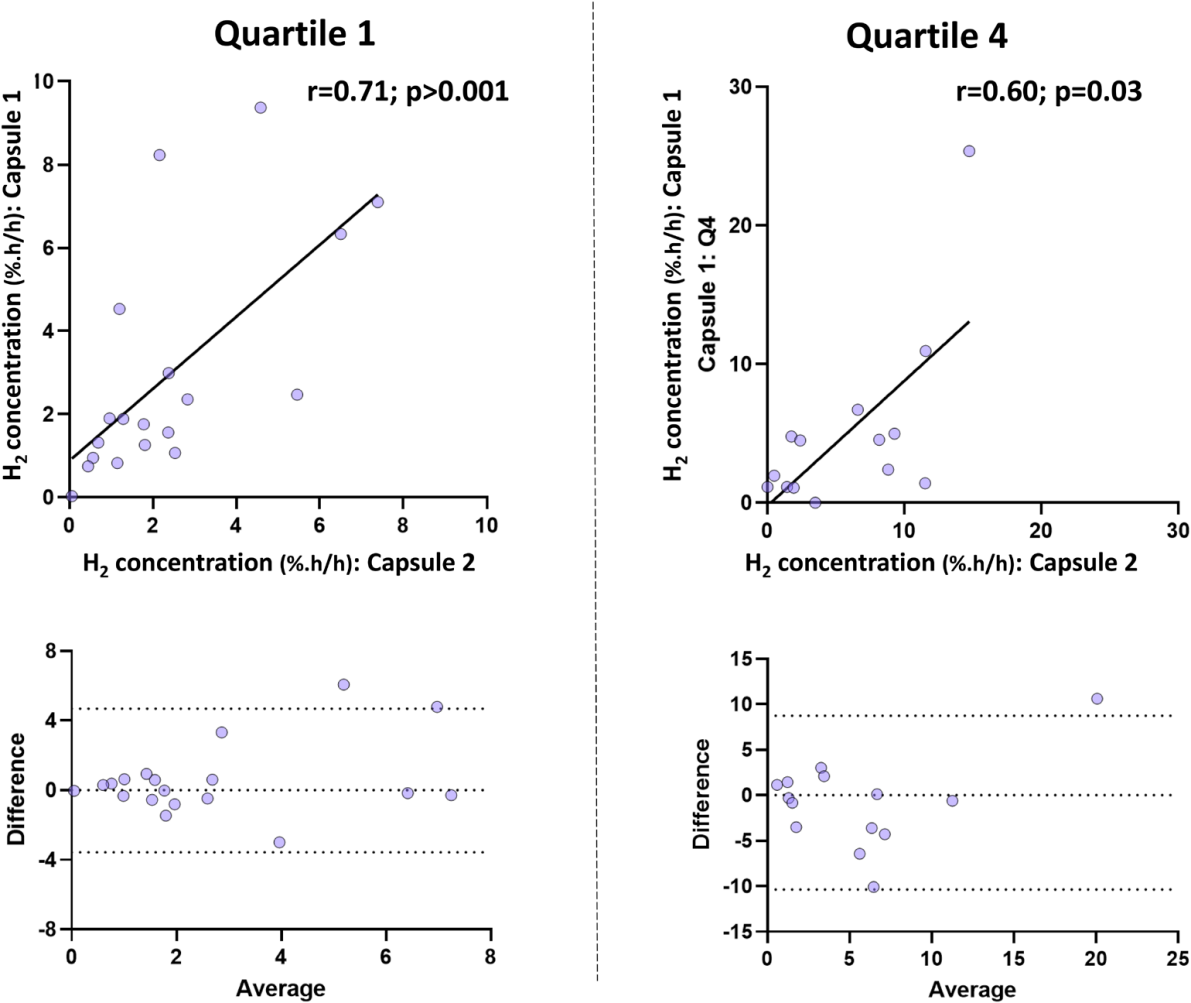
Comparison of hydrogen concentrations in the first (Q1) and fourth quartiles (Q4) (according to colonic transit) measured by tandemly ingested gas‐sensing capsules. Results are presented as Spearman correlations and as Bland–Altman plots, for which the bias was < 1% and the limits of agreement were −3.6 to 4.7 for Q1 and −10.4 to 8.8 for Q4.

**FIGURE 6:**
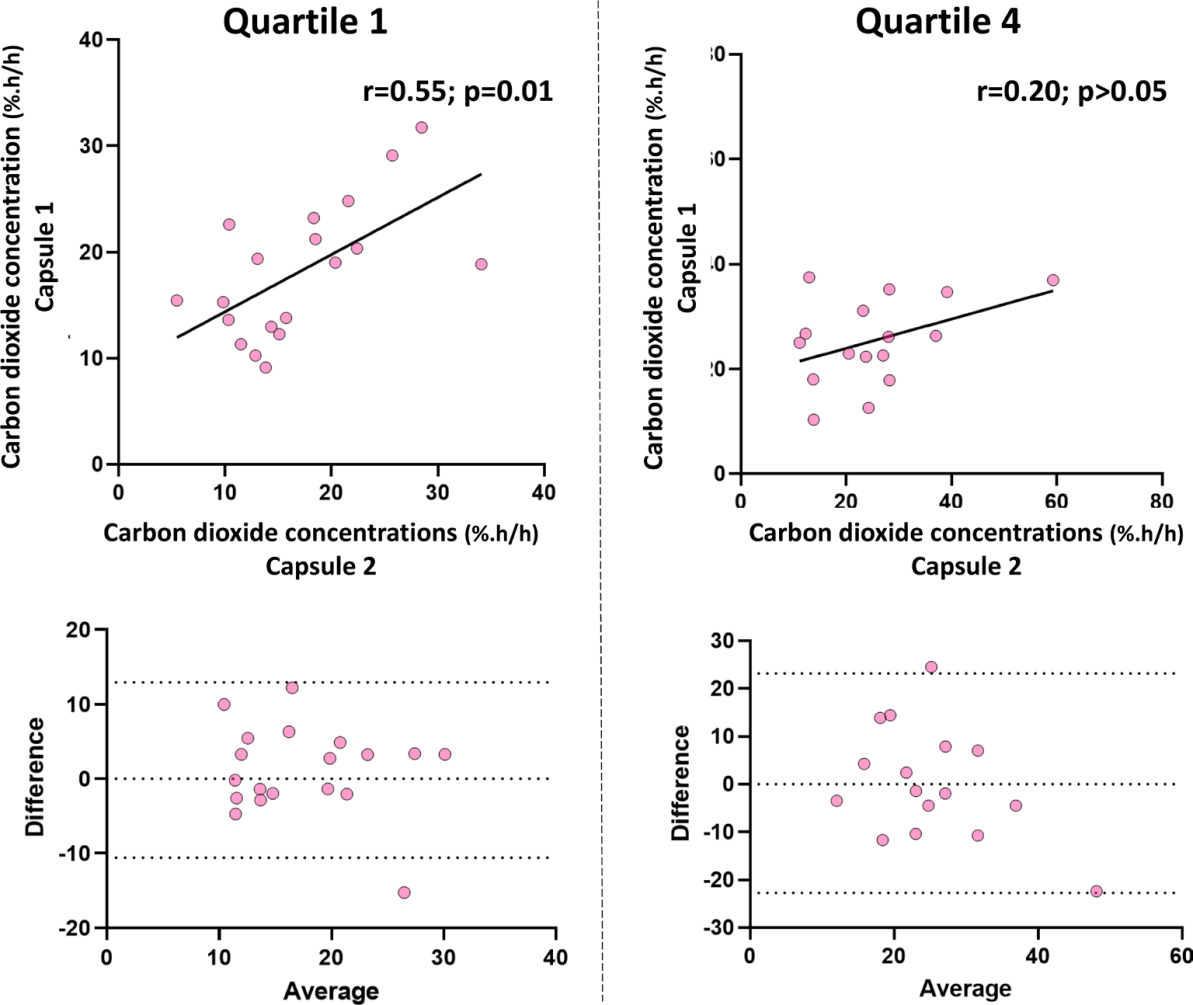
Comparison of carbon dioxide concentrations in the first (Q1) and fourth quartiles (Q4) (according to colonic transit) measured by tandemly ingested gas‐sensing capsules. Results are presented as Spearman correlations and as Bland–Altman plots, for which the bias was < 1.2% and the limits of agreement were −10.6 to 13.0 for Q1 and −22.7 to 23.2 for Q4.

From 24 participants simultaneously ingesting two gas‐sensing capsules, there were 20 evaluable pairs of data. Hydrogen concentrations between the tandem capsules were correlated in Q1 (*r*_*s*_ = 0.71, *p* < 0.001) and in Q4 (*r*_*s*_ = 0.60; *p* = 0.03; Figure 5). For carbondioxide, concentrations correlated in Q1 (*r*_*s*_ = 0.55, *p* = 0.01), but not in Q4 (*r*_*s*_ = 0.20, *p* > 0.05; Figure 6).

Bland–Altman plots showed minimal bias (hydrogen < 1.0 %.h/h; carbon dioxide < 1.2 %.h/h) between the two devices for both Q1 and Q4 (Figures 5 and 6). Paired data that were > 2 standard deviations of the bias were examined for potential confounders. There were 3 participants who recorded outlying results for hydrogen concentrations (*n* = 2 in Q1 only; *n* = 1 in Q4 only). In one of these participants, the rates of capsule transit through the colon differed by more than 50 h. For the other two outliers, colonic transit time were similar. Two subjects recorded outlying results for microbial metabolic activities (*n* = 1 in Q1 only; *n* = 1 in Q4 only). Capsule passage across the colon was similar between these capsules.

### 3.6 Repeat Ingestion of Gas‐Sensing Capsules

**FIGURE 7:**
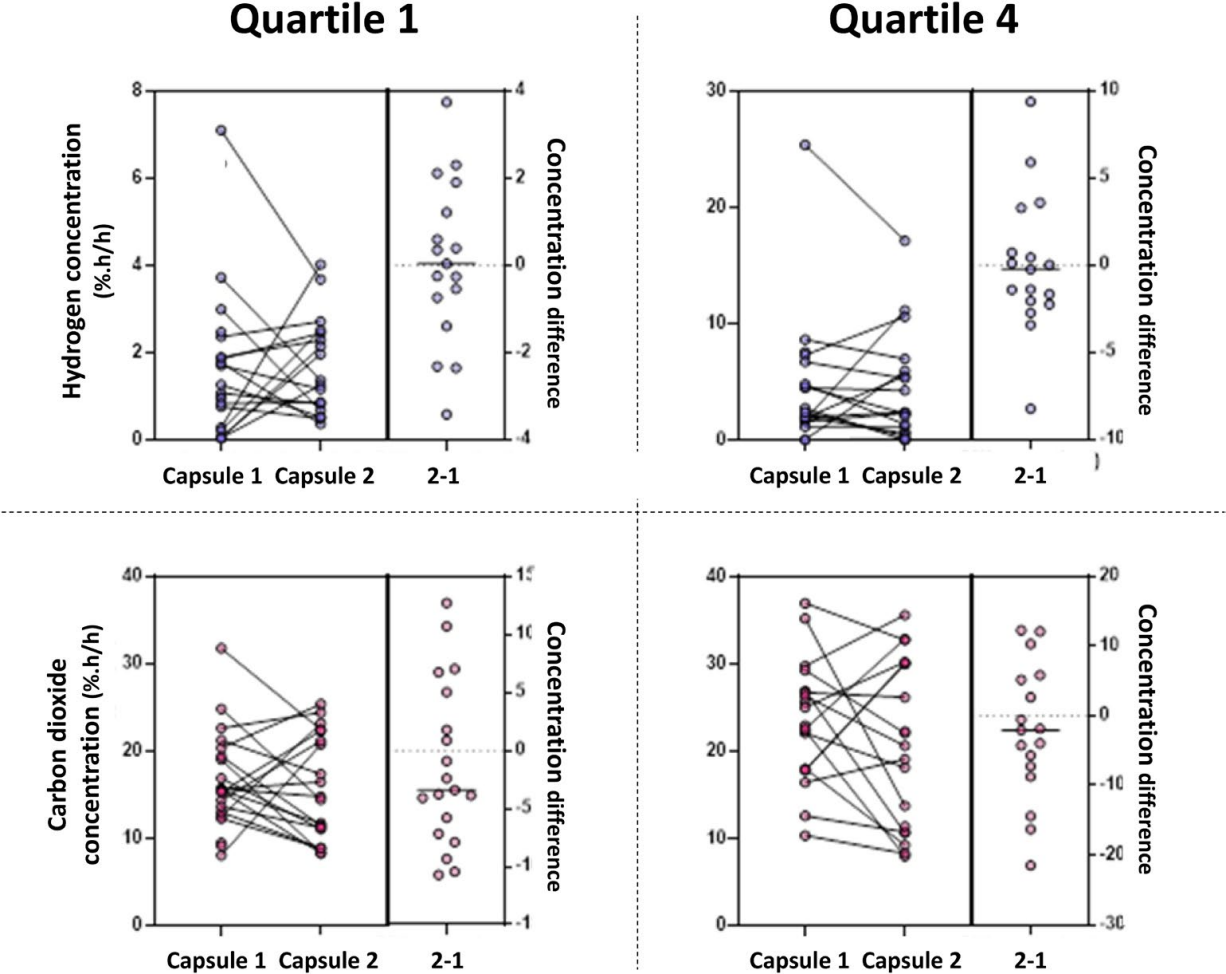
Comparison of concentrations of hydrogen and carbon dioxide via gas‐sensing capsule ingested on two different occasions according to Quartiles 1 and 4 (according to colonic transit). There were no statistically significant differences in gas metrics on repeat ingestion for hydrogen concentrations (Q1: *p* = 0.93; Q4: *p* = 0.78; Wilcoxon signed‐rank test) or for carbon dioxide concentrations (Q1: *p* = 0.37; Q4: *p* = 0.43).

There were no differences in gas metrics on repeat ingestion in Q1 and Q4 for hydrogen and carbon dioxide concentrations (Figure 7). Intra‐individual variance observed was smaller in magnitude in Q1 than in Q4: for hydrogen, variance was a median of 0 (−1.0–1.6) %.h/h for Q1 compared with −0.2 (−2.2–2.0) %.h/h for Q4, and for carbon dioxide, −3.4 (−7.2–5.0) %.h/h for Q1 compared with −2.1 (−8.0–5.5) %.h/h for Q4.

## 4 Discussion

The challenge in defining the dynamic and/or locoregional patterns of microbial activity, not just within colonic segments but along the entire gastrointestinal tract, may be overcome, at least in part, with the application of telemetric ingestible devices allowing real‐time, longitudinal assessment of the gastrointestinal tract [13]. In this study, we show that the combined use of pH‐sensing and gas‐sensing capsules can provide novel information about the spatial dynamics of fermentation by‐products at different segments of the colon for adult humans in an ambulatory, physiological state. The current study measured and compared the regional concentrations and patterns of hydrogen ions (pH), hydrogen gas, and carbon dioxide in healthy humans on an unrestricted, habitual diet. Key findings include that the three marker analytes measure different aspects of colonic physiology, and that the longitudinal profiles of these measures provide a more personalised assessment of gut microbial activity and function.

Given the novelty of gas measurement via sensing of luminal contents in vivo by the gas‐sensing capsule, the accuracy and reproducibility of the measurements were evaluated in two ways. First, the gas concentrations within the colon were compared between two capsules ingested in tandem (i.e., one immediately after the other). Indeed, the concentrations of hydrogen and carbon dioxide measured by these two gas‐sensing capsules were similar across the cohort in both the proximal and distal colon, strongly supporting the validity of the method of sampling and the measurements by the sensors. Previous analysis of data captured by the sensors within the gas‐sensing capsule confirmed that key gastrointestinal tract landmarks were similarly identified on tandem ingestion studies [10]. However, variance of the outputs of two gas‐sensing capsules within some individuals was noted, as also observed for transit measurements [10]. The variations observed were greater in the distal colon (Q4) compared with the proximal colon (Q1), which is perhaps not surprising for several reasons. First, the luminal contents become more solid in composition during transit from proximal to distal colon [3]. The degree of the faecal fluidity, influenced by colonic transit rate, alters the mixing of colonic contents and the partial pressures of the gases measured [10]. Second, transit time also can alter microbiota composition [16, 17]. Third, gases can be readily expelled as flatus from the distal colorectal segment/quartile and may alter the profile of the gases measured by the capsule [18]. Finally, the pattern of movement of the two capsules through the colon may differ, as grossly shown by differences in the timing of their exit from the bowel.

It is not possible currently to define the anatomical location of the capsule within the colon except when it first arrives (i.e., passes through the ileocaecal junction) and immediately prior to being expelled. Previous electromagnetic capsule tracking studies have identified various patterns of capsule movement, including fast, slow, retrograde, and antegrade as it travels along the colon [19]. With that in mind, colonic transit was divided into quartiles by time, and analysis of data concentrated on the proximal colon (Q1) and the distal colon (Q4) where position was more confidently localised. The gas profiles from the proximal and distal colon were similar for replicate capsule studies performed a median of 28 days apart in healthy individuals, with a variance that mimicked that seen in the tandem ingestion. The findings also reflected those reported for gastrointestinal transit times on repeated ingestions of gas‐sensing or pH‐sensing capsule at different time points [10, 20, 21]. From our previous demonstration that supplementation of fermentable and non‐fermentable fibres in a randomised cross‐over study led to consistent changes in the distribution of hydrogen concentrations [12, 22], it might have been anticipated that variations in the diet over time in the current participants would be associated with variation in distal colonic gas concentrations. However, the participants were given no specific instructions regarding their habitual diet, and so the results might be considered random assessment. The minimal variation in the summary results observed between repeat/replicate values is consistent with the stability of broad metabolic activity of colonic microbiota over the short term and presumed relatively stability of the participants' diets. Taken together, the results of the tandem and repeat studies did reassure us that the methodology employed for the gas‐sensing capsule was reliable and reproducible.

When comparing the distribution of the markers across the colon and between individuals, no correlation was found between luminal pH and the normalised concentrations of hydrogen or carbon dioxide in the proximal or distal colon. Luminal pH showed a proximal‐to‐distal ascending gradient in the majority of subjects (78%), in keeping with previous studies of healthy subjects [4, 23, 24]. A lower pH at the proximal colon most likely reflects the abundance of fermentable carbohydrate (both dietary and endogenous in origin) generating acidic by‐products, such as short‐chain fatty acids, principally acetate, propionate and butyrate, and perhaps lesser amounts of other organic acids, such as lactate and succinate [25, 26, 27, 28, 29]. Acidification of the proximal colonic lumen is countered by epithelial transport of bicarbonate to the lumen in response to uptake of short‐chain fatty acids by the epithelium [30]. Hence, luminal pH in the proximal colon is largely dictated by carbohydrate fermentation and the colonic epithelium's buffering capacity [30, 31]. In contrast, distal colonic fermentation is more variable and depends on carbohydrate substrate availability, as it is preferentially consumed as a source of energy over protein by microbes. Subsequently, protein and amino‐acid fermentation occur to a greater extent distally, yielding a different spectrum of by‐products, including phenolic and indolic compounds, weak acids, such as p‐cresol and branched‐chain fatty acids, and basic metabolic by‐products, including ammonia [4, 23, 31].

We found that carbon dioxide concentrations showed a similar proximal‐distal pattern to that of pH, with most subjects displaying greater concentrations in the distal colon compared with those in the proximal colon, but its concentrations did not correlate with luminal pH. The luminal carbon dioxide concentrations reflect the summation of its production and consumption/loss. Production of carbon dioxide occurs during both saccharolytic and proteolytic fermentation pathways and from bicarbonate‐mediated buffering, as well as through other metabolic pathways, such as the citric acid cycle and hydrolysis of urea and by diffusion across the epithelium into the lumen [32, 33]. Consumption of carbon dioxide can occur as part of microbial fermentation, for instance in the priming of propionate biosynthesis via the succinate pathway, and during acetogenesis by select bacteria or methanogenesis by archaea, in addition to its absorption across the epithelium and its from flatulence or exhalation [33, 34, 35, 36]. The carbon dioxide sensor also detects methane, but this can effectively be considered ‘carbon dioxide’ since it detects carbon dioxide and methane in a 1:1 ratio, and one molecule of carbon dioxide is converted to one molecule of methane by methanogens. The vast majority of these gases produced in the colon is due to microbial metabolic activity either generated directly from the microbes or by carbon dioxide production from bicarbonate‐related buffering of hydrogen ions from carbohydrate fermentation. The contribution of carbon dioxide diffusion from cellular metabolism in the tissues is unknown, but diffusion from tissues where carbon dioxide concentrations are less than 0.1% into the lumen where concentrations are greater than 10% would seem unlikely.

The production of hydrogen is almost solely derived from fermentation of carbohydrates [35], which mostly consist of dietary fermentable fibres and poorly absorbed sugars, with a smaller contribution from endogenous substrates, such as mucins [34, 36, 37, 38]. Because microbes preferentially derive energy from carbohydrate fermentation over that from protein fermentation, exhaustion of such substrates may occur distally in many individuals due to their consumption. Hydrogen generated from carbohydrate fermentation then faces multiple pathways of disposal. It may be lost via colonic epithelial/mucosal absorption and subsequent exhalation, or in flatus [36, 39]. Hydrogen can also be utilised by microbes via methanogenesis, homoacetogenesis, dissimilatory sulphate reduction by sulphate‐reducing bacteria, fumarate and nitrate reduction, and hydrogenation of unsaturated fatty acids [32, 33, 34]. The luminal pH affects the activity of hydrogen utilisation; methanogens and sulphate‐reducing bacteria are believed to function optimally at a neutral pH or in slightly alkaline conditions and thus distally in the colon, while acetogens are able to function in slightly acidic environments [32, 33, 34]. The more solid nature of the stool in the left colon relative to the right colon may also affect the gaseous partial pressure of hydrogen with increased hydrogenotrophic activity in the left colon due to reduced faecal stirring [40].

If it is assumed that the hydrogen‐cycling mechanisms exerted by the microbiota are roughly similar across the colon during the passage of the capsule, then regional variations in hydrogen concentrations measured will represent the differences in the relative amount of carbohydrate fermentation occurring and, therefore, reflect substrate availability. This notion is supported by the association of slower colonic transit with greater protein degradation and reduced faecal concentrations of short‐chain fatty acids [12], and by our observations in a feeding study of patients with irritable bowel syndrome, in which a descending proximal‐distal gradient in luminal hydrogen concentrations was converted to a rising gradient by supplementing the same diet with a fibre combination that increased the delivery of fermentable carbohydrates to the distal colon [21]. Our cohort of healthy subjects showed variation across individuals regarding the measured hydrogen concentrations and their proximal‐distal patterns (Figure 1) as might be anticipated with differences in diet, microbiota and associated disposal mechanisms. Confirmation that the proximal‐distal hydrogen gradient is a product of the types and amounts of fibre consumed requires further study.

One caveat to the interpretation of changing distal hydrogen concentrations as reflecting availability of carbohydrate substrate is the effect of methanogenesis. In this process, the generation of one methane molecule will utilise two hydrogen molecules, leading to a reduction in hydrogen concentration, whilst the carbon dioxide measure, as outlined above, will remain unchanged. Thus, falling distal hydrogen concentrations could potentially, at least in part, also reflect methanogenesis, which occurs in 28% of healthy adults [41] and most likely in the distal colon [42]. Since breath testing was not performed in our cohort, we were not able to identify the methane producers. An indirect marker of methanogenesis might be colonic transit, which is slower in methane producers [41]. While there was a correlation of colonic transit with carbon dioxide concentrations alone, this was evident in both proximal and distal segments and did not correlate with the ratio of carbon dioxide: hydrogen concentrations. With current methodologies, only targeted and interventional studies where methane production is modulated and methane excretion in the breath monitored could address this issue.

Slower transit through the proximal colon was associated with greater concentrations of hydrogen and carbon dioxide, presumably related to the time for fermentative and other metabolic functions to generate the gases, where substrates are plentiful. Luminal pH was not associated with transit time, presumably because buffering of hydrogen ion generation via bicarbonate secretion will increase in response to greater fermentation. In the distal colon, slower transit was also associated with greater carbon dioxide concentrations since this represents a longer duration of microbial metabolic activity. By contrast, hydrogen concentrations do not correlate with the transit time through the distal colon since its production is likely to be dictated by substrate availability, which will be influenced by both dietary fibre intake and transit. Luminal pH increased in association with slower transit, most likely a function of greater protein fermentation with release of basic metabolites, such as ammonia. However, many assumptions are made for these explanations and will require further study including interventions to vary substrate availability.

### 4.1 Strengths and Limitations

The strengths of the current study include the assessment of the precision of the measurements made in tandem gas‐sensing capsule experiments, the size of the cohort, and that several participants underwent a repeat ingestion at a separate time point. Another strength of the study was that the ingestible wireless motility capsules are also capable of measuring transit time, a factor that can influence (and be influenced by) fermentation, substrate, and water availability [41]. Additionally, in our interpretation of the results, we did not assume that the capsule moved across the colon in a consistent manner and, therefore, focused on examining the findings in proximal (Q1) and distal (Q4) segments, which should be reliable as they were associated with entry and exit from the colon, respectively.

However, there are several limitations. First, there is a lack of gold standard, since these gas measurements were breaking new ground in the gastrointestinal tract physiology assessment, which has been a ‘black‐box’ environment until now. Second, the inability of the thermal conductivity sensor to distinguish between carbon dioxide and methane implies that methane concentrations cannot be quantified. This limitation may have clinical relevance due to the gasotransmitter effects of methane on motility and confounds the interpretation of the hydrogen concentrations if methane production varies in its location in the colon. Third, interpretation of intra‐ and inter‐individual heterogeneity would have been enhanced by concurrent dietary assessment.

### 4.2 Potential Clinical Application

There is considerable potential clinical application of the locoregional assessment of fermentation in the colon with gas‐sensing capsule beyond the ability to measure regional transit times that are validated and FDA‐cleared. The belief that regional colonic fermentation is a key to preventive and therapeutic health of the colon has been prevalent for a long time and has been an integral part of standard clinical gastroenterological practice. For example, the manipulation of fibre intake has been a common therapy applied in several conditions [43]. These include the treatment of constipation to enhance colonic transit, the amelioration of functional gut symptoms, the delivery of SCFA to the distal colon for their anti‐inflammatory and anticarcinogenic effects in the management of ulcerative colitis and prevention of colorectal cancer. The evidence for such use is limited for many reasons. These include the inability to measure the target of the fibre‐based therapy both in understanding the physiology that is being manipulated and monitoring whether the fibre is having the desired effect. Furthermore, the classification of fibres according to their actions within the gastrointestinal tract has only recently emerged from less informative chemical definitions according to solubility in water [44]. Hence, fibre is currently used in a trial‐and‐error approach. The gas‐sensing capsule offers to be able to disrupt such practice by measuring key targets in ambulant people without bowel preparation, and with relatively minimal inconvenience. The targets are regional and whole gut transit times and regional carbohydrate fermentation. There is preliminary evidence that patients with irritable bowel syndrome may benefit in the choice of fibre supplement by measurement of transit times and proximal‐distal hydrogen concentrations with the outcomes of improved gastrointestinal function and uniformity of SCFA delivery to the mucosa of the entire colon [12, 21]. Moreover, the success of the intervention can be monitored by repeat testing with the gas‐sensing capsule. Since this is the dawn of a new era, the outcomes of such personalised fibre usage await further evaluation. The limited information about the transit and fermentation patterns in patients with other conditions, such as inflammatory bowel disease, precludes speculation of the clinical value at the present time. However, it is likely that our understanding of many gastrointestinal diseases will be enhanced by novel data on the nature of physiological disturbances and how therapeutic manipulations affect them.

## 5 Conclusion

The results of this study highlight that the gas‐sensing wireless motility capsule provides novel insight into the dynamic metabolic processes of the colon, including fermentation. While luminal pH measured by the pH‐sensing capsule might offer a broad perspective of fermentation, we suggest that the gas‐sensing capsule provides more detailed information, including overall microbial metabolic activity via carbon dioxide concentrations and hydrogen concentration representing a more specific measure of carbohydrate fermentation. We introduce the concept of quartile ratio assessment to define the proximal‐distal distribution of fermentation in the colon. Future work is required to understand the role of methane production in interpreting the results, the importance of these fermentation profiles in longitudinal studies, and whether ‘unbalanced’ profiles can be changed with intervention leading to measurable changes in patient health and wellbeing.

## Author Contributions

**Phoebe A. Thwaites:** conceptualization, investigation, methodology, validation, visualization, writing – original draft, writing – review and editing, formal analysis, project administration, data curation. **Chu K. Yao:** investigation, methodology, writing – review and editing. **Jasjot Maggo:** investigation, methodology, writing – review and editing. **Francis C. Parker:** methodology, formal analysis, writing – review and editing. **Emma P. Halmos:** investigation, writing – review and editing. **Rebecca E. Burgell:** investigation, writing – review and editing. **Jane G. Muir:** investigation, writing – review and editing. **Daniel So:** methodology, formal analysis, data curation, writing – review and editing. **Kourosh Kalantar‐Zadeh:** conceptualization, methodology, formal analysis, writing – review and editing, data curation. **Richard B. Gearry:** investigation, funding acquisition, writing – review and editing, supervision. **Peter R. Gibson:** conceptualization, methodology, formal analysis, data curation, supervision, funding acquisition, writing – review and editing.

## Conflicts of Interest

J.G.M., F.C.P., J.G.M.: no competing interests. P.A.T.: Has received conference sponsorship from Pfizer. C.K.Y.: Received research support from Atmo Biosciences for investigator‐initiated studies and travel honoraria from Viatris, Yakult Australia and Dr. Falk Pharma. R.E.B.: Consultant or advisory board member for Allergan, Atmo Biosciences, Antara. She has received speaking honoraria from Bayer. D.S.: has received consulting and speaking honoraria from Procter & Gamble, and received research support from, and is a current employee of Atmo Biosciences. E.P.H.: was supported through research grants from the National Health & Medical Research Council Investigator Grant and Crohn's & Colitis Foundation Litwin Pioneers Program. E.P.H. has also received research grants from Mindset Health Pty Ltd., Intoleran Pty Ltd. and Gastroenterological Society of Australia IBD Clinical Project award. She has received honoraria or consulted for Ferring, Janssen, Abbvie, Takeda, Shire, Sandoz & Dr. Falk Pharm. K.K.Z.: advisory board member for Atmo Biosciences. RBG: received research support from Atmo Biosciences, Janssen, AbbVie, Pfizer, Comvita, Zespri. Speaker fees from AbbVie, Takeda, Janssen, Pharmaco, Zespri. Advisory boards for Janssen, Takeda, Celltrion, AbbVie, Zespri. P.R.G.: consultant or advisory board member for Anatara, Atmo Biosciences, Topas and Comvita; research grants for investigator‐driven studies from Mindset Health, and speaker honoraria from Dr. Falk Pharma and Mindset Health Pty Ltd. Shareholder in Atmo Biosciences. J.G.M., P.R.G., C.K.Y., E.P.H. and J.E.V. work in a department that financially benefits from the sales of a digital application (Monash University FODMAP diet app), patient booklets, cookbooks and online courses all of which relate to the low FODMAP diet therapy. These commercial activities now contribute to the salaries of J.G.M., C.K.Y. and P.R.G.

## Supporting information


**Figure S1:** Individual results in participants having valid data from the pH‐sensing and gas‐sensing capsules ingested in tandem according to the quartiles (Q) of colonic transit.
**Table S1:** Data loss from the gas‐sensing capsule overall and according to quartile of colonic transit. Measures with a loss of data > 50% were excluded.

## Data Availability

The data that support the findings of this study are available from the corresponding author, [PRG], upon reasonable request.
