# Supplementary material for: Telemetric Assessment and Comparison of Regional Colonic Metabolic Activity in Ambulant Healthy Individuals Using pH and Gas‐Sensing Wireless Motility Capsules
Source: Aliment Pharmacol Ther. 2025 Aug 26;63(2):242–53. doi: 10.1111/apt.70345 (PMC12746393; doi:10.1111/apt.70345)

**SUPPLEMENTARY INFORMATION**

**Supplementary Table 1.** Data loss from the gas-sensing capsule overall and according to quartile of colonic transit. Measures with a loss of data >50% were excluded

| **Gas** | **Segment**  **assessed** | **Excluded**  **Number** | **Included** | |
| --- | --- | --- | --- | --- |
|  |  |  | Number | Median data loss, % |
| **Hydrogen** | Overall | 7 | 103 | 18.2 |
|  | Quartile 1 | 4 | 106 | 14.5 |
|  | Quartile 2 | 12 | 98 | 17.1 |
|  | Quartile 3 | 14 | 96 | 15.8 |
|  | Quartile 4 | 13 | 97 | 17.1 |
| **Carbon dioxide** | Overall | 7 | 103 | 20.0 |
|  | Quartile 1 | 2 | 108 | 12.5 |
|  | Quartile 2 | 9 | 101 | 16.7 |
|  | Quartile 3 | 11 | 99 | 13.1 |
|  | Quartile 4 | 15 | 95 | 16.7 |

**Supplementary Figure 1.** Individual results in participants having valid data from the pH-sensing and gas-sensing capsules ingested in tandem according to the quartiles (Q) of colonic transit. **A)** pH that differed across quartile (p<0.0001; Friedman’s test); **B)** hydrogen (H_2_) concentrations that differed across quartiles (p<0.013); **C)** carbon dioxide (CO_2_) concentrations that differed across quartiles (p<0.0001); and **D)** CO_2_:H_2_ ratios where differences across quartile were no statistically significant (p=0.28).


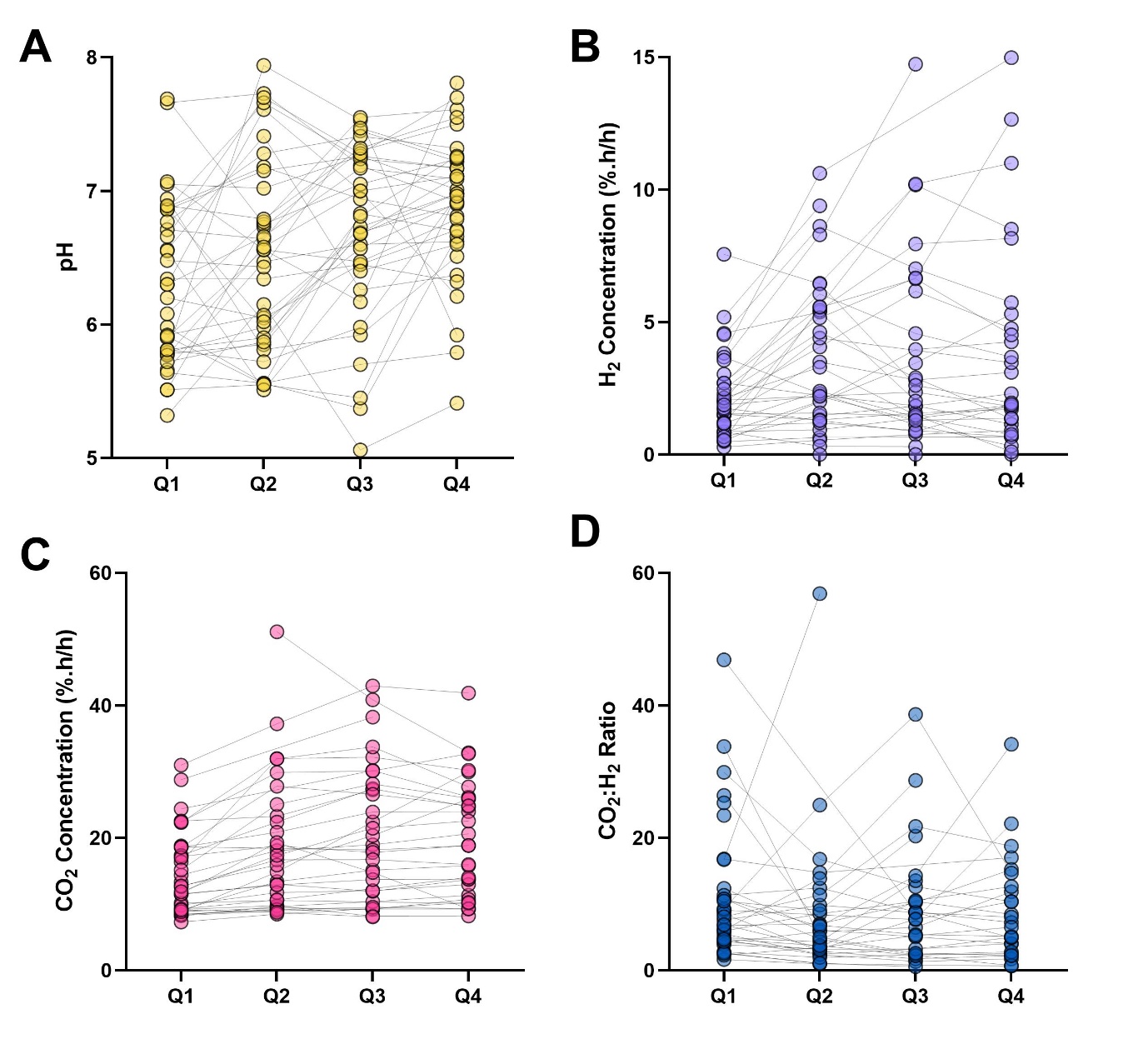

Supplement: Supplementary file 1 — Figure S1: Individual results in participants having valid data from the pH‐sensing and gas‐sensing capsules ingested in tandem according to the quartiles (Q) of colonic transit. Table S1: Data loss from the gas‐sensing capsule overall and according to quartile of colonic transit. Measures with a loss of data > 50% were excluded. [file APT-63-242-s001.docx]
